# Analysis of PANoptosis-related ceRNA network reveals lncRNA MIR17HG involved in osteogenic differentiation inhibition impaired by tumor necrosis factor-α

**DOI:** 10.1007/s11033-024-09810-0

**Published:** 2024-08-15

**Authors:** Jia-Xuan Li, Yu-Dun Qu, Chang-Liang Xia, Wei Zhang, Song-Song Wang, Shuan-Ji Ou, Yang Yang, Yong Qi, Chang-Peng Xu

**Affiliations:** 1 The Affiliated Guangdong Second Provincial General Hospital of Jinan University, No. 466 Xingang Road, Haizhu District, Guangzhou, 510317 Guangdong People’s Republic of China; 2 Department of Orthopaedics, Southern Medical University, Guangzhou, Guangdong China; 3 School of Medicine, XiaMen University, Xiamen, Fujian China

**Keywords:** PANoptosis, lncRNA, Osteoblasts, TNF-α, Osteomyelitis, Bibliometrics, Bioinformatics

## Abstract

**Background:**

Inflammatory cytokines such as Interleukin 1β(IL1β), IL6,Tumor Necrosis Factor-α (TNF-α) can inhibit osteoblast differentiation and induce osteoblast apoptosis. PANoptosis, a newly identified type of programmed cell death (PCD), may be influenced by long noncoding RNA (lncRNAs) which play important roles in regulating inflammation. However, the potential role of lncRNAs in inflammation and PANoptosis during osteogenic differentiation remains unclear. This study aimed to investigate the regulatory functions of lncRNAs in inflammation and apoptosis during osteogenic differentiation.

**Methods and results:**

High-throughput sequencing was used to identify differentially expressed genes involved in osteoblast differentiation under inflammatory conditions. Two lncRNAs associated with inflammation and PANoptosis during osteogenic differentiation were identified from sequencing data and Gene Expression Omnibus (GEO) databases. Their functionalities were analyzed using diverse bioinformatics methodologies, resulting in the construction of the lncRNA-miRNA-mRNA network. Among these, lncRNA (MIR17HG) showed a high correlation with PANoptosis. Bibliometric methods were employed to collect literature data on PANoptosis, and its components were inferred. PCR and Western Blotting experiments confirmed that lncRNA MIR17HG is related to PANoptosis in osteoblasts during inflammation.

**Conclusions:**

Our data suggest that TNF-α-induced inhibition of osteogenic differentiation and PANoptosis in MC3T3-E1 osteoblasts is associated with MIR17HG. These findings highlight the critical role of MIR17HG in the interplay between inflammation, PANoptosis, and osteogenic differentiation, suggesting potential therapeutic targets for conditions involving impaired bone formation and inflammatory responses.

**Supplementary Information:**

The online version contains supplementary material available at 10.1007/s11033-024-09810-0.

## Introduction

Inflammatory cytokines play pivotal roles in the pathogenesis of bone infections. Tumor Necrosis Factor-alpha (TNF-α) is a key pro-inflammatory cytokine and a catabolic factor in the inflammatory response to disease [1, 2]. Persistent inflammation significantly affects bone regeneration as TNF-α binds to different receptors and initiates signal transduction pathways [3, 4], leading to various cellular responses including cell survival, differentiation, and proliferation. Once the signal transduction pathways are elucidated, strategies can be adopted to reduce the inhibitory effects of inflammation on tissue regeneration [5, 6]. Among the various signal transducer and activator of transcription (STATs), STAT3 is particularly important in mediating inflammation and bone formation [7–9]. Our previous study showed that under TNF-α stimulation, Elongator acetyltransferase complex subunit 2 (ELP2) inhibits osteogenic differentiation through STAT3 activation [10].

In an inflammatory environment, the differentiation capacity of osteoblasts is inhibited, and osteoblast death is exacerbated [11, 12]. Osteoblasts often undergo apoptosis in response to inflammation, but other forms of programmed cell death (PCD) mechanisms such as pyroptosis and necroptosis also play crucial roles [13, 14]. Although each of these three PCD pathways has been previously regarded as unique based on the identification of important promoters, effectors, and executors, there is mounting evidence that they interact extensively [15, 16]. Based on these findings, we developed the concept of panoptosis. It is an inflammatory PCD route controlled by the PANoptosome complex, which possesses important characteristics of pyroptosis, apoptosis, and/or necroptosis. These characteristics could not be explained by any of the PCD pathways alone [17–19].

The miR-17-92 cluster, encoded by the host gene MIR17HG, is involved in various biological activities, including tumor development, neurodevelopment, and immune regulation [20–22]. This cluster has a regulatory role in inflammation [23], with studies showing that inhibition of MIR17HG can attenuate neuronal apoptosis and inflammatory responses in Parkinson’s disease (PD) mice [24]. Recent research has linked MIR17HG to osteoarthritis [22], suggesting its involvement in other bone diseases such as osteomyelitis and osteoarthritis, where inflammation is a key factor.

To identify new diagnostic and therapeutic approaches for bone infections, this study aims to investigate the relationship between MIR17HG and molecular processes involved in inflammation during bone infections. Using cell sequencing and the Gene Expression Omnibus (GEO) database, we identified distinct Long non-coding RNA (lncRNA) linked to osteogenic differentiation and PANoptosis under TNF-α stimulation. We constructed lncRNA-miRNA-mRNA networks and performed bioinformatics analyses to explore their functions further. Furthermore, we investigated the correlation between the expression of lncRNA MIR17HG and mRNAs associated with PANoptosis in osteoblasts in response to TNF-α stimulation. This research introduces a novel framework to understand the mechanisms responsible for PANoptosis onset in osteoblasts triggered by TNF-α stimulation.

## Methods and materials

### Reagents and antibodies

Minimum essential medium α (MEM‐α) and fetal bovine serum (FBS) obtained from Bio-channel (Nanjing, China). Dexamethason, β‐glycerophosphate and ascorbic acid were purchased from Sigma Chemicals (St. Louis, MO, USA). Trypsin was obtained from Gibco. TRIzol™ reagents were purchased from Thermo Fisher Scientific (Waltham, MA, USA). Recombinant Mouse TNF alpha(Cat. No.: CF09), NovoStart®SYBR qPCR SuperMix Plus and NovoScript®Plus All-in-one 1st Strand cDNA Synthesis SuperMix (gDNA Purge) were obtained from Novorprotein (Suzhou, China). Primary and secondary antibody for Western blot (WB) experience, including STAT3 (D3Z2G), P-Phospho-Stat3(Tyr705) IgG(L27A9), and GAPDH (14C10) antibodies were purchased from Cell Signaling Technology (Boston, MA, USA).

### RNA sequencing analysis

#### Cell culture

The murine pre-osteoblast MC3T3-E1 clonal cell line was purchased from iCell Bioscience (Shanghai, China). Cells were cultured in MEM -α with 10% FBS in a humidified environment with 5% CO_2_ at 37 ℃. The medium was supplemented with penicillin, glutamine, streptomycin, and sodium pyruvate. For osteogenic differentiation, 10 mM glycerophosphate and 50 μg/ml ascorbic acid were added at the end of the logarithmic phase.

#### RNA isolation and sequencing

Prior to RNA isolation, MC3T3-E1 cells were cultured in six-well plates for 7 days as described. They were then divided into two groups of six independently cultured samples each: one treated with TNF-α (5 ng/ml) for 24 h and the other cultured without treatment for the same duration. RNA was isolated by scraping the cells and using Trizol reagent (ThermoFisher, Shanghai, China). Evaluation of RNA samples included: (1) agarose gel electrophoresis to analyze RNA degradation and contamination; (2) Nanodrop to determine RNA purity (OD260/280 ratio); (3) Qubit for precise RNA concentration quantification; (4) Agilent 2100 for RNA integrity assessment (Novogene, Beijing, China). The RNA samples were then sent to Beijing Novogene Technology Co., LTD for sequencing analysis.

### Data collection

#### Bioinformatics data collection

Data on the differential expression of osteogenic differentiation markers in osteoblasts under inflammatory conditions were obtained using an MC3T3-E1 inflammation model. This model included samples from the inflammation group (TNF-α = 5 ng/ml) and the osteogenic differentiation group (TNF-α = 0 ng/ml). Genes associated with normal osteogenic differentiation were obtained from the GSE30393 transcriptomic dataset (Genomic characterization of osteoblastic differentiation of MC-3T3E1 cells) in the GEO database https://www.ncbi.nlm.nih.gov/geo/query/acc.cgi?acc=GSE30393). Differentially expressed genes were identified for both the inflammation model (criterion: |Log2 Fold Change (FC)|≥ 0.75, P < 0.0001) and the GSE30393 dataset (criterion: |Log2 FC|≥ 0.5, P < 0.05) using the limma package (version 3.52.4) in R. Eleven PANoptosis-associated genes, mainly PANoptosome components[25], were extracted based on previous studies. PANoptosis-associated lncRNAs were identified using a Pearson correlation coefficient of |R|> 0.7 and significance P < 0.05.

#### Bibliometric approach

Bibliometric analysis is a convenient and accurate tool for discovering hot spots and trends in specific research areas [26, 27]. We selected Web of Science (https://www.webofscience.com) as the target database and employed three search strategies: (1) TOPIC (Pyropotosis) AND TOPIC (Apoptosis) AND TOPIC (Necropotosis); (2) TOPIC (PANoptosis); (3) TOPIC (PANoptosome). We conducted keyword visualization analysis on the literature data collected from the three search strategies and specifically analyzed the literature data related to PANoptosomes using bibliometric software tools such as EXCEL 2023 and VOSviewer 1.6.19. VOSviewer 1.6.19 investigates trends in specific research directions and noteworthy scientific publications, and we used it to create a keyword co-occurrence map and then cluster all keywords. We used Microsoft Office Excel 2023 to manage the data and investigate publication trends.

### Methods of bioinformatics analysis

#### Screening for PANoptosis and inflammation-related lncRNA in osteomyelitis

The VennDiagram R package (version 1.7.3) was used to analyze the dataset and identify potential lncRNA candidates. The Pheatmap R package (version 1.0.12) was used to create a heatmap showing the expression of two potential lncRNAs, PANoptosis-related mRNAs, and normal osteogenic differentiation markers. Candidate lncRNAs were further characterized using the R package ggplot2 (version 3.4.1). The relationship between potential lncRNAs and PANoptosis-associated genes was examined using the R package ggplot2 (version 3.4.1).

#### Construction of lncRNA-miRNA-mRNA networks

DIANA Tools—lncBase V.3 (https://dianalab.e-ce.uth.gr/tools) was used to project miRNA targets by combining PANoptosis-related lncRNAs. We utilized the MicroRNA Target Prediction Database (miRDB) database (http://mirdb.org/index.html) to forecast the mRNA targets of the identified miRNAs. Pearson’s correlation coefficient analysis (P < 0.05, statistically significant) was used to predict co-expressed miRNAs and mRNA targets with lncRNAs. Cytoscape 3.10.1 software was employed to construct and visualize the lncRNA-miRNA-mRNA network.

#### Analysis of mRNAs' functional enrichment in ceRNA networks

We performed functional enrichment analysis on a total of 2415 mRNAs that were co-expressed with lncRNAs in the lncRNA-miRNA-mRNA network. The R package clusterProfiler was used to conduct Kyoto Encyclopedia of Genes and Genomes (KEGG) and Gene Ontology (GO) analyses.

### Construction of MC3T3-E1 inflammation model

MC3T3-E1 cells were seeded into six-well plates at a density of 2 × 10^5^ cells/mL. Once the cells reached 70% confluence, they were cultured in osteoblast differentiation medium for 0–14 days, and the medium (2 mL) was changed every two days. The osteoblast differentiation medium consisted of MEM-α with 10% FBS, 0.1 μg/ml dexamethasone, 10 μg/ml glycerol phosphate, and 50 μg/ml ascorbic acid. After 14 days, cells were exposed to TNF-α (0, 5 ng/ml, 50 ng/ml) for 24 h in an incubator before further analysis.[28, 29] Based on our group's previous studies, we found that the onset of PANoptosis and osteogenic differentiation inhibition was more pronounced in MC3T3-E1 cells stimulated with 50 ng/ml of TNF-α. Therefore we added this concentration to the inflammation model we constructed.

### Real-time fluorescence quantitative PCR (RT-qPCR) analysis

TRIzol reagent (Vazyme, Nanjing, China) was used to extract total RNA from MC3T3-E1 cells according to the manufacturer’s instructions. Total RNA was reverse transcribed into cDNA using a reverse transcription kit (Takara, Beijing, China). Real-time fluorescence quantitative PCR was performed on a LightCycler480II (Roche, Basel, Switzerland). MIR17HG and other primer sequences are listed in Supplement Table 1. Quantitative primer sequences were collected from the Primer Design Tool (https://www.ncbi.nlm.nih.gov/tools/primer-blast/index.cgi) and Primer Bank (https://pga.mgh.harvard.edu/primerbank/index.html).

**Table 1:** Correlation analysis of MIR17HG and 4930447F24Rik with PANoptosis-related mRNAs

Id	Correlation_pearson	P value_pearson	Correlation_spearman	P value_spearman
Casp1	1	0	1	0
Mir17hg	0.732559911	0.006734918	0.629370629	0.032394750
4930447F24Rik	0.940467298	0.000005327	0.734265734	0.009052097
Casp3	1	0	1	0
Mir17hg	−0.730175809	0.007009470	−0.475524476	0.121319356
Casp7	1	0	1	0
Mir17hg	0.888432478	0.000112571	0.678321678	0.018825126
Casp8	1	0	1	0
Mir17hg	0.705797205	0.010322481	0.468531469	0.127472653
4930447F24Rik	0.951518868	0.000001944	0.818181818	0.002027199
Casp9	1	0	1	0
Mir17hg	−0.897892799	0.000073477	−0.790209790	0.003616814
Fadd	1	0	1	0
Mir17hg	0.769773887	0.003409462	0.601398601	0.042806869
4930447F24Rik	0.666668691	0.017899649	0.601398601	0.042806869
Gsdmd	1	0	1	0
Mir17hg	0.802869480	0.001666223	0.769230769	0.005252921
Mlkl	1	0	1	0
Mir17hg	0.889885822	0.000105694	0.762237762	0.005897476
Ripk1	1	0	1	0
Mir17hg	0.792914943	0.002093671	0.741258741	0.008170640
4930447F24Rik	0.901227217	0.000062599	0.825174825	0.001718596
Ripk3	1	0	1	0
Mir17hg	0.930975623	0.000010981	0.839160839	0.001191874
4930447F24Rik	0.864904411	0.000281241	0.755244755	0.006596543

### Western blotting

MC3T3-E1 cells treated with TNF-α were lysed with RIPA lysis buffer containing a protease inhibitor cocktail (Thermo Fisher Scientific, Victoria, Australia). After centrifugation at 12,000 rpm for 20 min at 4 ℃, the supernatant was collected. Protein concentration was determined using the BCA Protein Assay Kit in accordance with the manufacturer’s instructions. Proteins were separated by SDS-PAGE, transferred to PVDF membranes, and blocked with 5% skimmed milk for 1 h at room temperature. Membranes were incubated with the primary antibody overnight at 4 °C, followed by a peroxidase secondary antibody for 1 h. Proteins were detected using an enhanced chemiluminescence kit (Millipore, Billerica, MA, USA) according to the manufacturer’s instructions.

### Statistical analysis

Data are represented as the mean ± standard deviation. Group differences were examined using Student’s t-tests. Pearson’s correlation coefficient was used to evaluate the relationships between variables. Data analysis was performed using R (version 4.3.1), with P < 0.05 considered statistically significant.

## Results

### Identification of inflammation-related and PANoptosis-related lncRNA in osteogenic differentiation

**Fig. 1:**
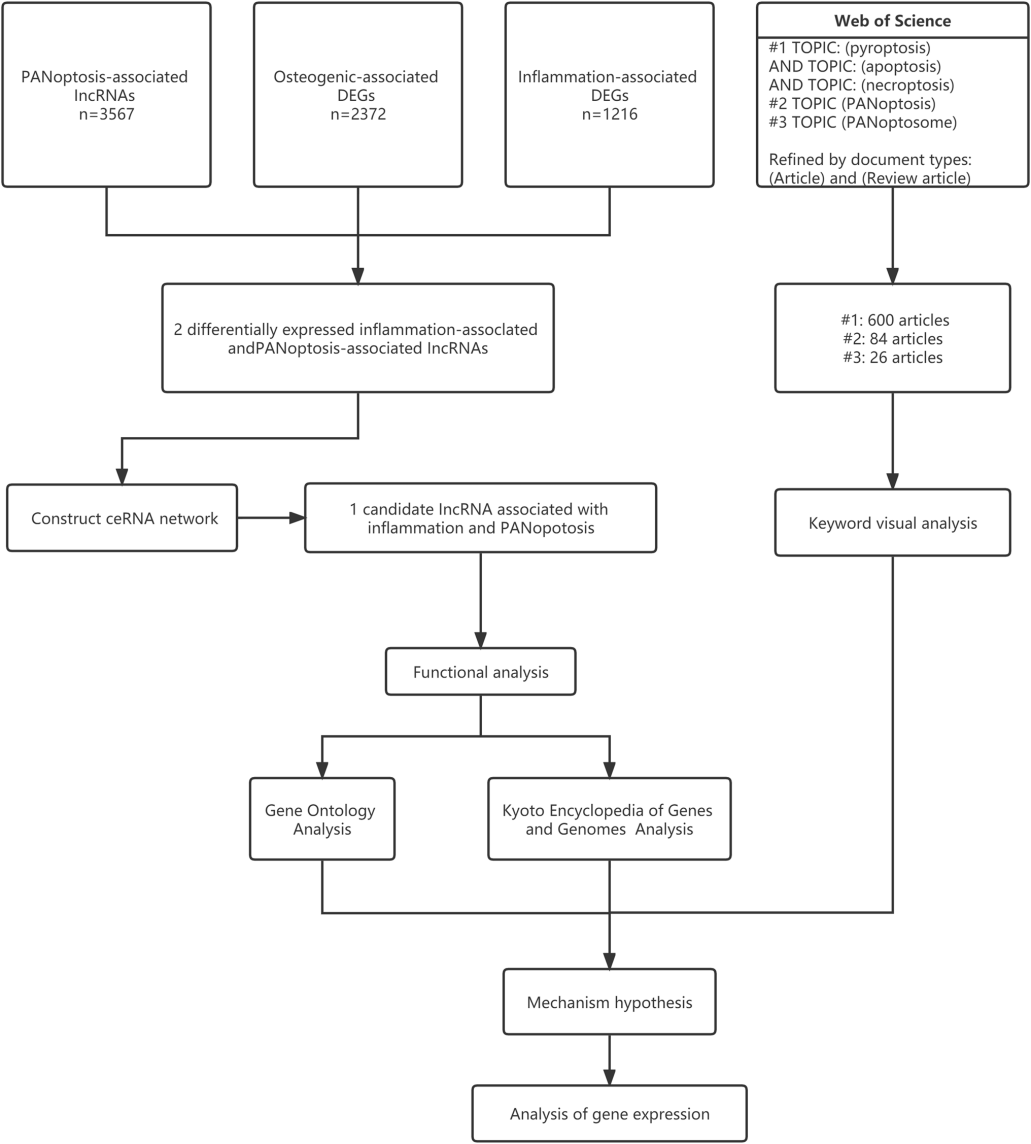
Workflow diagram

**Fig. 2:**
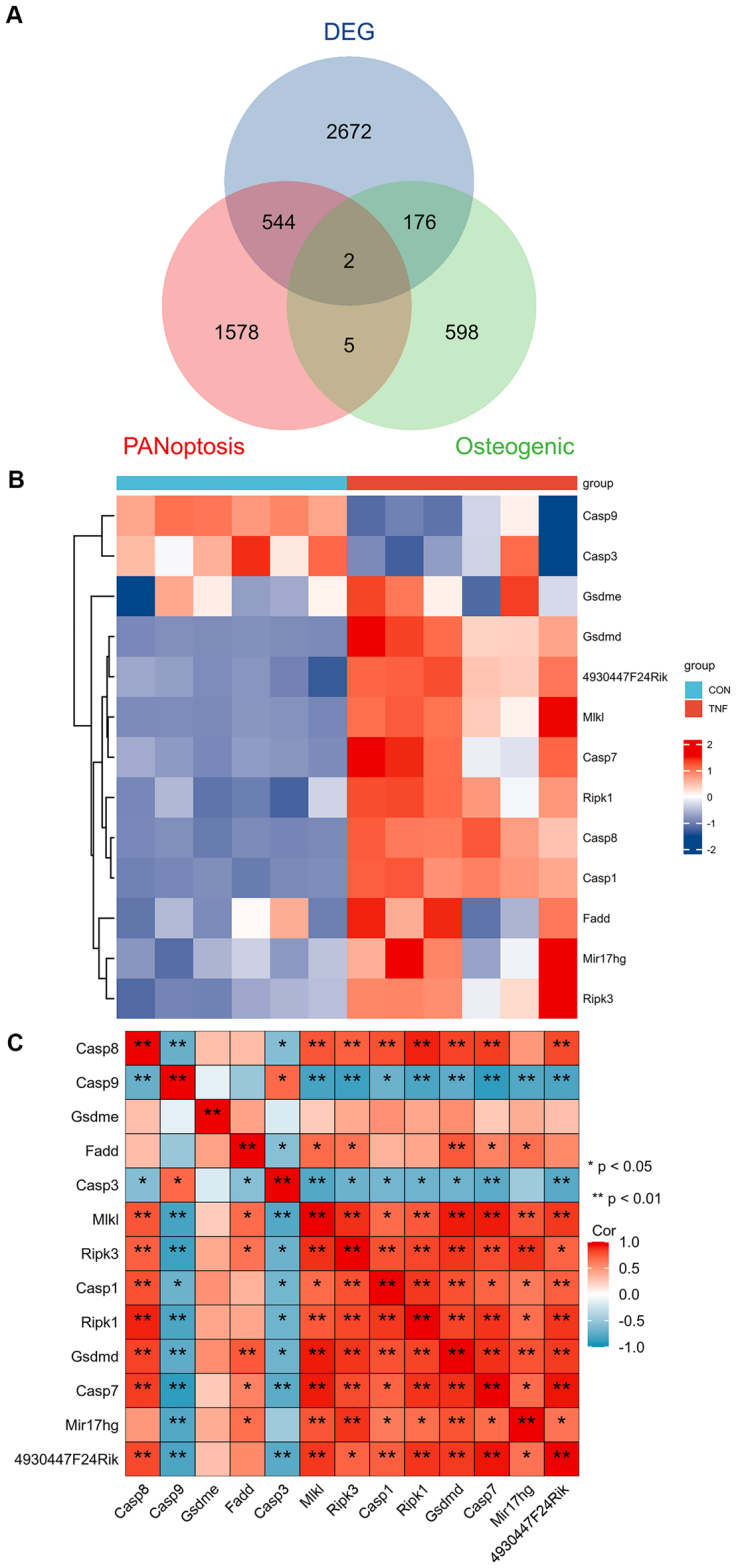
Identification of osteogenesis and PANoptosis-related lncRNAs. **A** Venn diagram identifying TNF- α stimulation related genes, PANoptosis-related lncRNAs and differentially expressed genes for osteogenesis. **B** Heatmap showing differential expression of 2 candidate lncRNAs (MIR17HG and 4930447F24Rik) and PANoptosis key genes in TNF- α stimulation related and osteogenesis. **C** Correlation heatmap showing the correlation between 2 candidate lncRNAs (MIR17HG and 4930447F24Rik) and PANoptosis-associated mRNAs

Figure 1 shows our research workflow diagram. We built an osteoblast inflammatory model for high-throughput sequencing, allowing us to obtain expression matrices. Through this sequencing, we identified 3721 differentially expressed genes (|Log2 FC |≥ 0.75, P < 0.0001). We retrieved transcriptome profiles for normal osteogenic differentiation from the GEO database (GSE30393). We identified differentially expressed genes in GSE30393 (|Log2 FC|≥ 0.5, P < 0.05). From literature review, we identified 11 genes associated with PANoptosis: CASP1, CASP3, CASP7, CASP8, CASP9, Gasdermin E(GSDME), Gasdermin D(GSDMD), MLKL, RIPK1, RIPK3, and FADD[25]. We conducted single-gene correlation analysis for these genes using |R|> 0.7 and P < 0.05 to identify PANoptosis-associated lncRNAs, based on data from high-throughput sequencing of inflammation models. MIR17HG and 4930447F24Rik were present in all three groups (Fig. 2A) and correlated with most PAN-associated genes. Heat maps showed the expression levels of MIR17HG and 4930447F24Rik in inflammation and osteogenic differentiation samples (Fig. 2B). Expression of MIR17HG and 4930447F24Rik was higher in the inflammation model than in normal osteogenically differentiated cells. The correlation heatmap showed that MIR17HG and 4930447F24Rik were associated with most panoptosis-related mRNAs (Fig. 2C). In correlation analyses, MIR17HG was associated with at least nine mRNAs, whereas 4930447F24Rik was associated with five mRNAs (Table 1).

### The lncRNA-miRNA-mRNA network construction

**Fig. 3:**
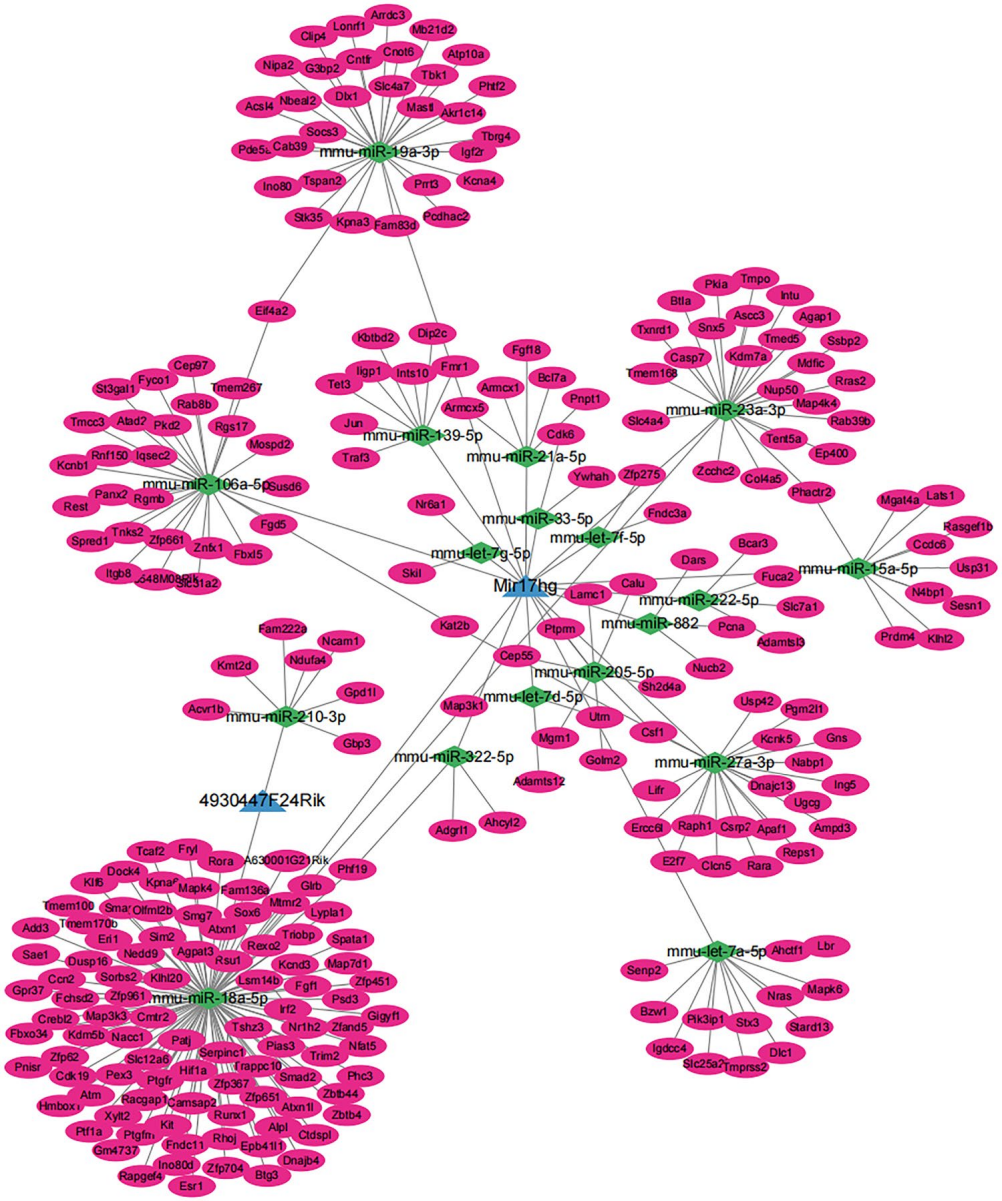
Construction of ceRNA Network of Candidate lncRNAs (MIR17HG and 4930447F24Rik)

To investigate the function of candidate lncRNAs in osteogenic differentiation under PANoptosis and inflammation conditions, we created an lncRNA-miRNA-mRNA network (Fig. 3). Using DIANA LncBase v.3 database, we identified miRNA targets interacting with potential lncRNA targets. We evaluated mRNA targets of miRNAs (Target Score ≥ 80) using the miRDB database v6.0. We identified 18 miRNAs and 263 mRNAs centered on candidate lncRNAs (Table 2) and the lncRNA-miRNA-mRNA network visualisation is shown in Fig. 3 (Cytoscape 3.10.1). The ceRNA map showed that MIR17HG is centrally positioned.

**Table 2:** lncRNAs, miRNAs and mRNAs in the ceRNA network

lncRNAs	Binding miRNAs	Associated mRNAs
MIR17HG	mmu-miR-106a-5p, mmu-miR-18a-5p, mmu-miR-205-5p, mmu-miR-222-5p, mmu-miR-882, mmu-let-7d-5p, mmu-let-7f-5p, mmu-let-7 g-5p, mmu-miR-139-5p, mmu-miR-15a-5p, mmu-miR-19a-3p, mmu-miR-21a-5p, mmu-miR-23a-3p, mmu-miR-27a-3p, mmu-miR-33-5p, mmu-miR-322-5p, mmu-let-7a-5p	Panx2, Iqsec2, Znfx1, Tmem267, Fyco1, Zfp661, Eif4a2, Fbxl5, Rgs17, Tnks2, 6430548M08Rik, Tmcc3, Kat2b, Susd6, Slc31a2, Kcnb1, Pkd2, St3gal1, Rest, Atad2, Cep97, Mospd2, Spred1, Fgd5, Rab8b, Itgb8, Rnf150, Rgmb, Hif1a, Irf2, Cep55, Golm2, Mgrn1, Calu, Csf1, Ptprm, Lamc1, Sh2d4a, Adamtsl3, Slc7a1, Bcar3, Fuca2, Nucb2, Pcna, Dars, Utrn, Adamts12, Zfp275, Fndc3a, Skil, Nr6a1, Dip2c, Iigp1, Traf3, Fmr1, Ints10, Jun, Kbtbd2, Tet3, Sesn1, Usp31, Phactr2, Ccdc6, Mgat4a, Prdm4, Lats1, N4bp1, Rasgef1b, Klhl2, Pcdhac2, Slc4a7, Acsl4, Stk35, Arrdc3, G3bp2, Lonrf1, Cntfr, Tspan2, Nipa2, Prrt3, Cnot6, Socs3, Kpna3, Atp10a, Dlx1, Igf2r, Tbk1, Kcna4, Mastl, Ino80, Fam83d, Clip4, Cab39, Tbrg4, Akr1c14, Pde5a, Phtf2, Mb21d2, Nbeal2, Armcx5, Armcx1, Fgf18, Pnpt1, Bcl7a, Kdm7a, Agap1, Btla, Txnrd1, Tmpo, Zcchc2, Slc4a4, Tent5a, Col4a5, Snx5, Map4k4, Casp7, Pkia, Map3k1, Nup50, Ssbp2, Rras2, Tmed5, Tmem168, Ep400, Mdfic, Rab39b, Intu, Ascc3, Apaf1, Raph1, Reps1, Dnajc13, Rara, Clcn5, Ampd3, Nabp1, Csrp2, Ugcg, Lifr, Kcnk5, Ing5, Pgm2l1, Gns, Usp42, Ercc6l, E2f7, Cdk6, Ywhah, Ahcyl2, Phf19, Adgrl1, Stard13, Dlc1, Igdcc4, Nras, Tmprss2, Mapk6, Bzw1, Ahctf1, Slc25a27, Senp2, Lbr, Stx3, Pik3ip1
4930447F24Rik	mmu-miR-18a-5p, mmu-miR-210-3p	Klhl20, Phc3, Esr1, Trappc10, Rora, Hif1a, Sorbs2, Psd3, Irf2, Atxn1, Pias3, Eri1, Nfat5, Runx1, Zfp367, Cdk19, Gpr37, Ptgfrn, Pnisr, Fgf1, Tmem170b, Slc12a6, Smad2, Zfp62, Nr1h2, Patj, Sim2, Map3k1, Serpinc1, Kpna6, Rsu1, Glrb, Nacc1, Tmem100, Spata1, Crebl2, Fchsd2, Tcaf2, Zfp704, Sox6, Dnajb4, Phf19, Fam136a, Nedd9, Gigyf1, Rexo2, Cmtr2, Lypla1, Zbtb4, Mtmr2, Camsap2, Kdm5b, A630001G21Rik, Mapk4, Ptf1a, Zfp651, Tshz3, Zfand5, Ccn2, Dusp16, Map7d1, Pex3, Trim2, Epb41l1, Atxn1l, Zfp961, Kcnd3, Kit, Fbxo34, Ino80d, Rhoj, Sae1, Dock4, Rapgef4, Alpl, Racgap1, Smap2, Add3, Fndc11, Fryl, Ctdspl, Hmbox1, Btg3, Klf6, Xylt2, Zbtb44, Gm4737, Smg7, Map3k3, Zfp451, Lsm14b, Olfml2b, Triobp, Atm, Agpat3, Ptgfr, Ncam1, Acvr1b, Gpd1l, Kmt2d, Fam222a, Ndufa4, Gbp3

### The functional enrichment analysis of mRNAs associated with MIR17HG.

**Fig. 4:**
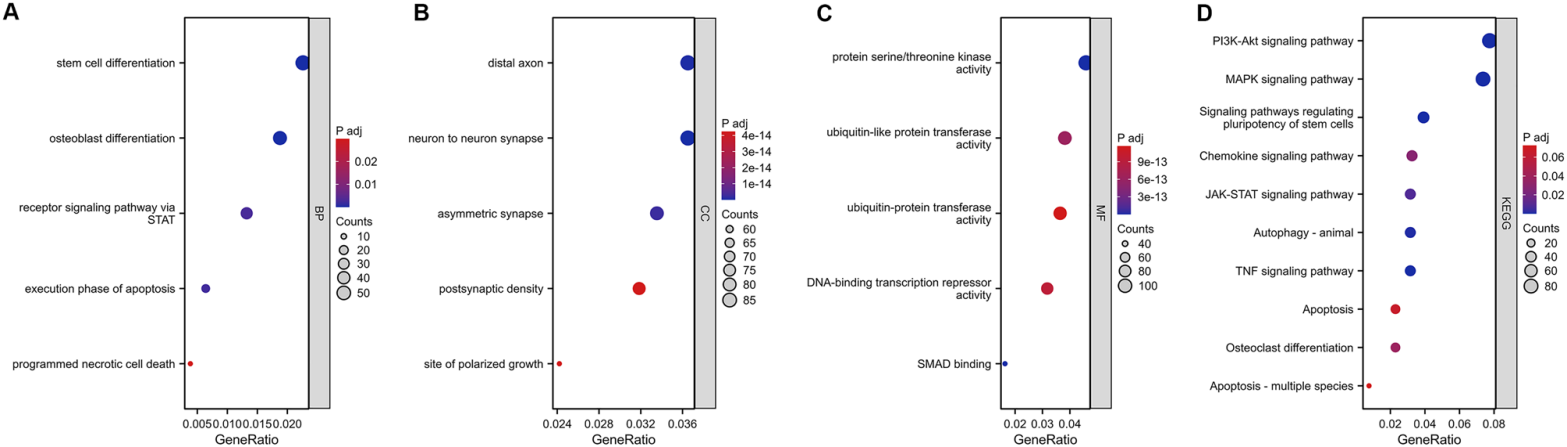
Functional and pathway enrichment analysis of mRNAs in MIR17HG-associated mRNAs (**A**–**D**). **A**–**C** shows the GO analysis of mRNAs in MIR17HG-related mRNAs, and **D** show the KEGG analysis of MIR17HG-related mRNAs

The strong correlation of MIR17HG with most PANoptosis-related genes is shown in Table 1. Given the relatively weaker correlation between 4930447F24Rik and PANoptosis-related genes, and MIR17HG’s central position in the ceRNA map, we performed functional enrichment analysis on MIR17HG-associated mRNAs. GO and KEGG analyses were conducted on 2415 lncRNA-associated mRNAs (Target Score ≥ 60) to investigate biological activities and signaling pathways of potential lncRNA (Fig. 4). The GO analysis covered biological processes (BP), cellular components (CC), and molecular functions (MF). BP included stem cell differentiation, osteoblast differentiation, apoptosis, and receptor signaling via STAT. CC included neuron-to-neuron synapse, distal axon, asymmetric synapse, site of polarized growth, and postsynaptic density. MF included protein serine/threonine kinase activity, SMAD binding, DNA-binding transcription repressor activity, and ubiquitin-protein transferase activity. KEGG analysis showed associations with MAPK, PI3K-Akt, TNF, JAK-STAT signaling pathways, autophagy, and osteoclast differentiation. GO and KEGG analysis results are in Supplementary Tables 2 and 3 (P < 0.05).

### Bibliometric analysis and mechanism hypothesis

**Fig. 5:**
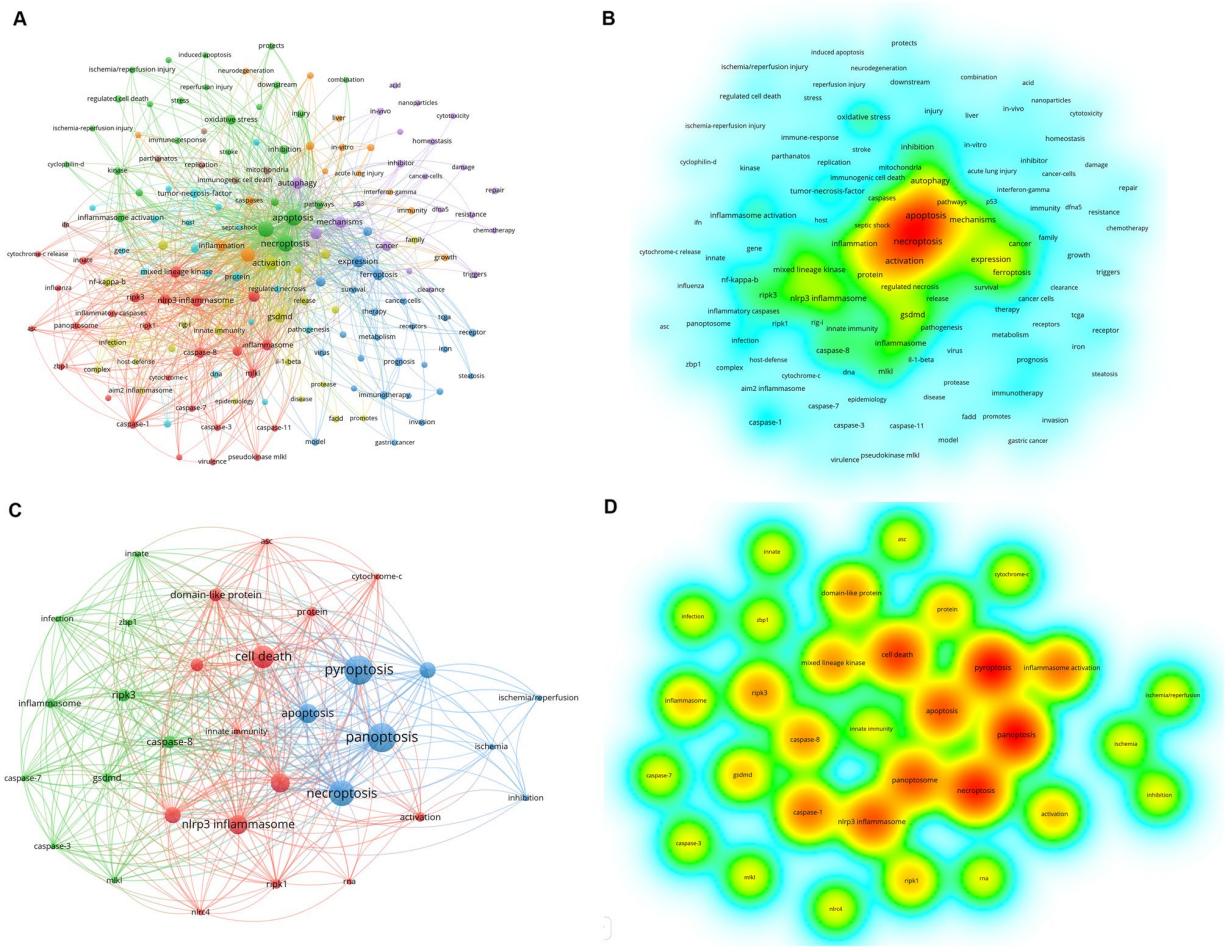
Network visualization of keywords (**A**&**B**, T = 5; **C**&**D**, T = 3). **A**&**B** shows the network diagram and thermal diagram for visual analysis of PANoptosis-related keywords, and **C**&**D** shows the network diagram and thermal diagram for visual analysis of PANoptosis-related keywords. The size of each circle represents the weight of the keyword. The distance between two circles indicates the correlation between the two circles. The stronger the correlation, the shorter the distance. The color of the circle indicates the corresponding cluster class. The color of the thermal diagram from green to yellow indicates a higher frequency of occurrence

Using visual keyword analysis, we performed bibliometric analysis. We obtained 241, 47, and 12 literature records from three search strategies. After removing duplicates, we conducted keyword visualization analysis (Fig. 5). The results highlighted frequent mentions of CASP1, 3, 7, 8, GSDMD, MLKL, RIPK1, 3, ZBP1, and NLRP3 in PANoptosis literature. Based on bioinformatics and bibliometric analyses, we propose a hypothesis for MIR17HG regulation of PANoptosis: Under TNF-α stimulation, MIR17HG expression increases in MC3T3-E1 osteoblasts, regulating miRNA transcription from the miR-17-92 cluster, which activates STAT3 and its phosphorylation. STAT3 activation, in turn, increases MIR17HG expression. Phosphorylated STAT3 regulates the downstream NLRP3 inflammasome via the STAT3 signaling pathway, signaling to the panoptosome and triggering PANoptosis.

### The role of MIR17HG and miR-17–92 cluster in PANoptosis

**Fig. 6:**
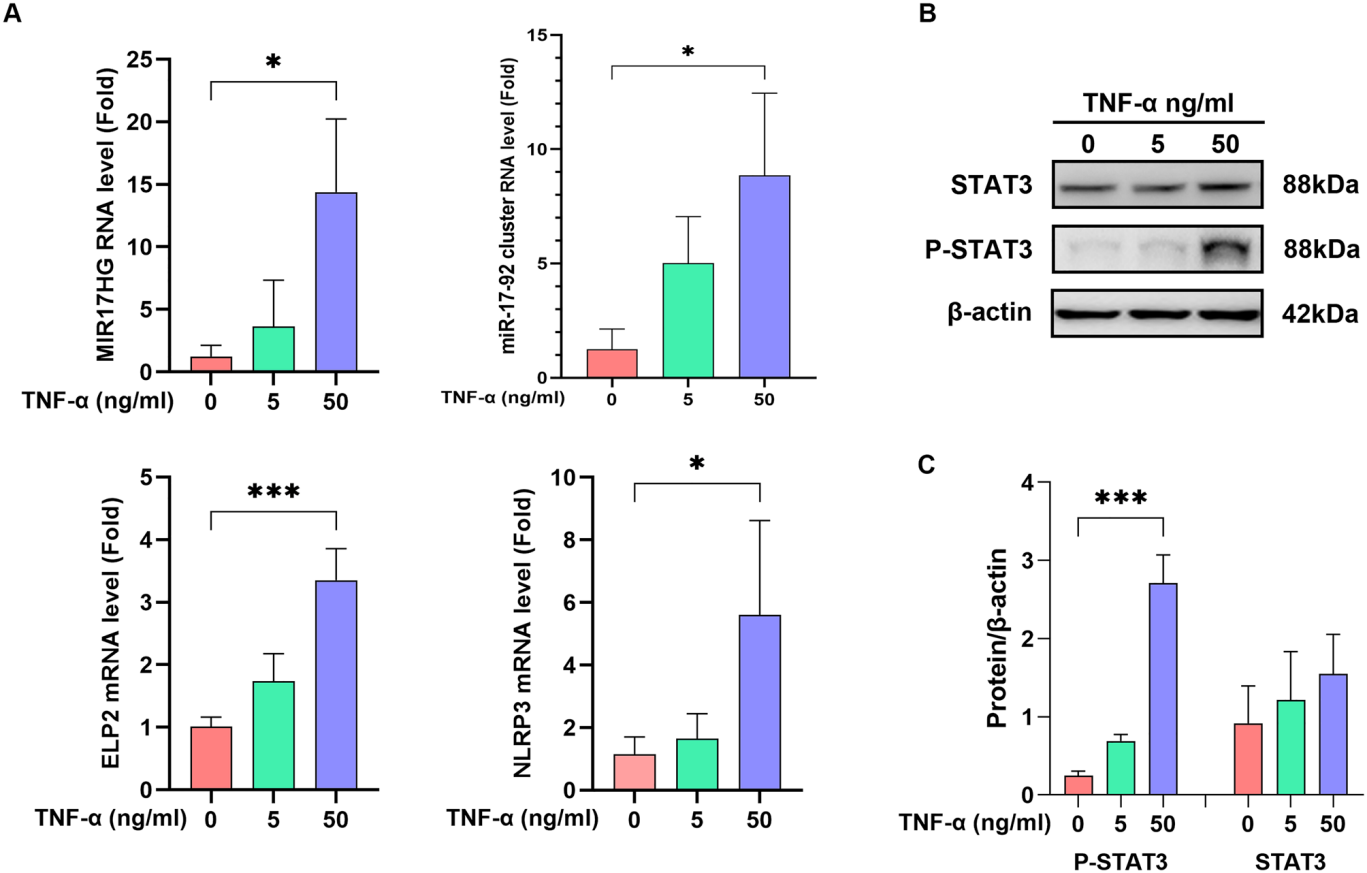
MIR17HG, miR-17-92 cluster, ELP2, and NLRP3 expression in response to TNF-α stimulation (**A**). phosphorylation of STAT3 after TNF-α stimulation was detected by Western blotting analysis (**B**&**C**)

**Fig. 7:**
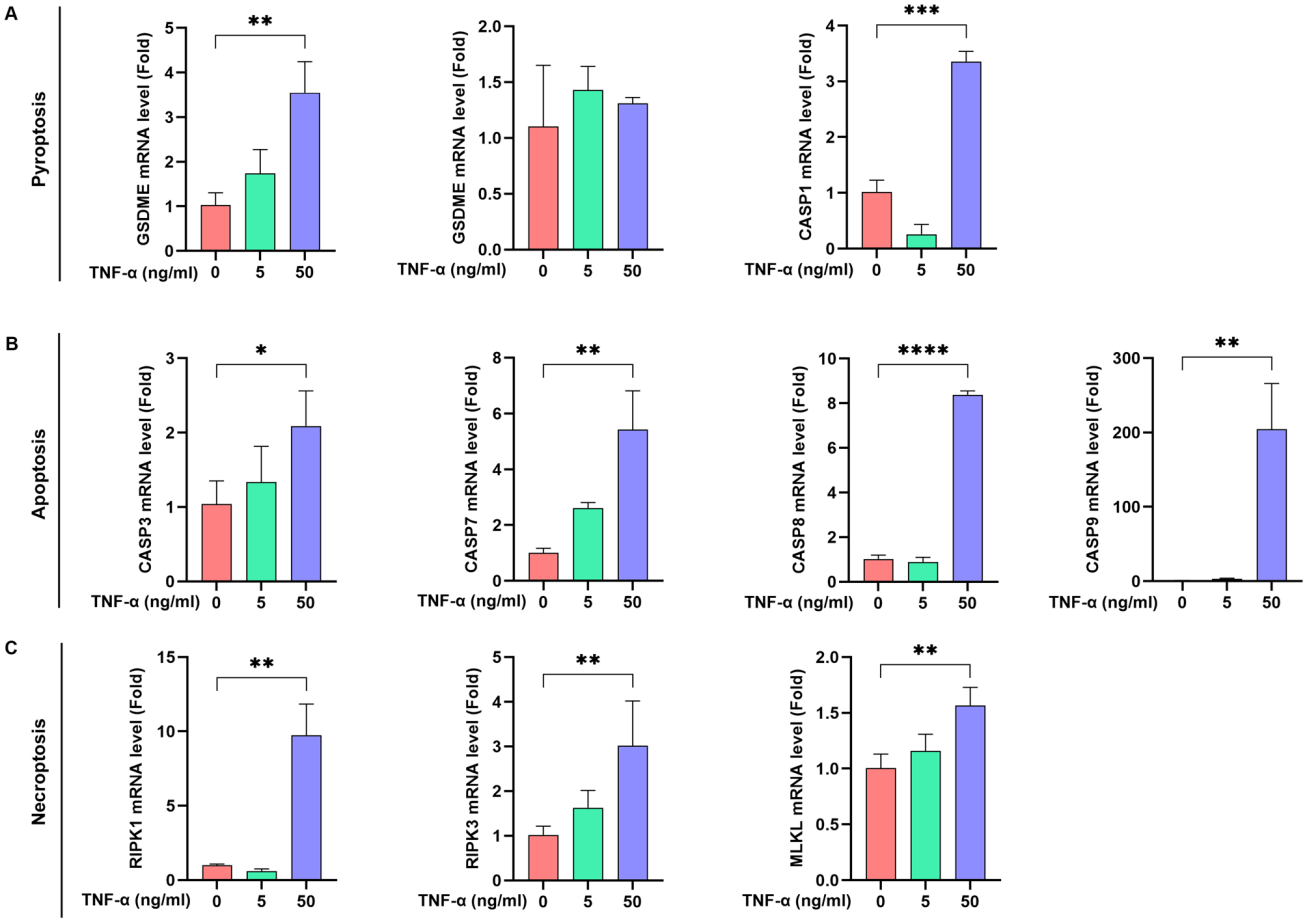
Expression of PANoptosis-related genes in response to TNF-α stimulation. Pyroptosis-related genes (**A**), apoptosis-related genes (**B**), and necroptosis-related genes (**C**)

To verify the correlation between MIR17HG expression and PANoptosis, MC3T3-E1 cells were treated with TNF-α for 24 h at 7 days of induction of differentiation and compared to the control group (no stimulation). And 5 ng/ml and 50 ng/ml TNF-α concentrations were chosen to stimulate MC3T3-E1 cells according to our previous study [28, 29]. RNA expression of MIR17HG, ELP2, NLRP3, and PANoptosis genes was detected by RT-PCR. Protein expression of STAT3 and its phosphorylation (p-STAT3) were detected by western blot analysis. As shown in Fig. 6A, RNA expression of MIR17HG and miR-17-92 clusters increased with TNF-α concentration. ELP2 and NLRP3 showed similar behavior. Figure 6B and C showed no significant change in STAT3 protein expression, but p-STAT3 increased TNF-α concentration. PANoptosis-related genes also showed an up-regulation trend (Fig. 7). These results suggest a positive correlation between MIR17HG levels and PANoptosis, with MIR17HG and miR-17-92 clusters regulating STAT3 phosphorylation and affecting PANoptosis occurrence.

### TNF-α stimulation’s effect on osteogenic differentiation and inflammation

**Fig. 8:**
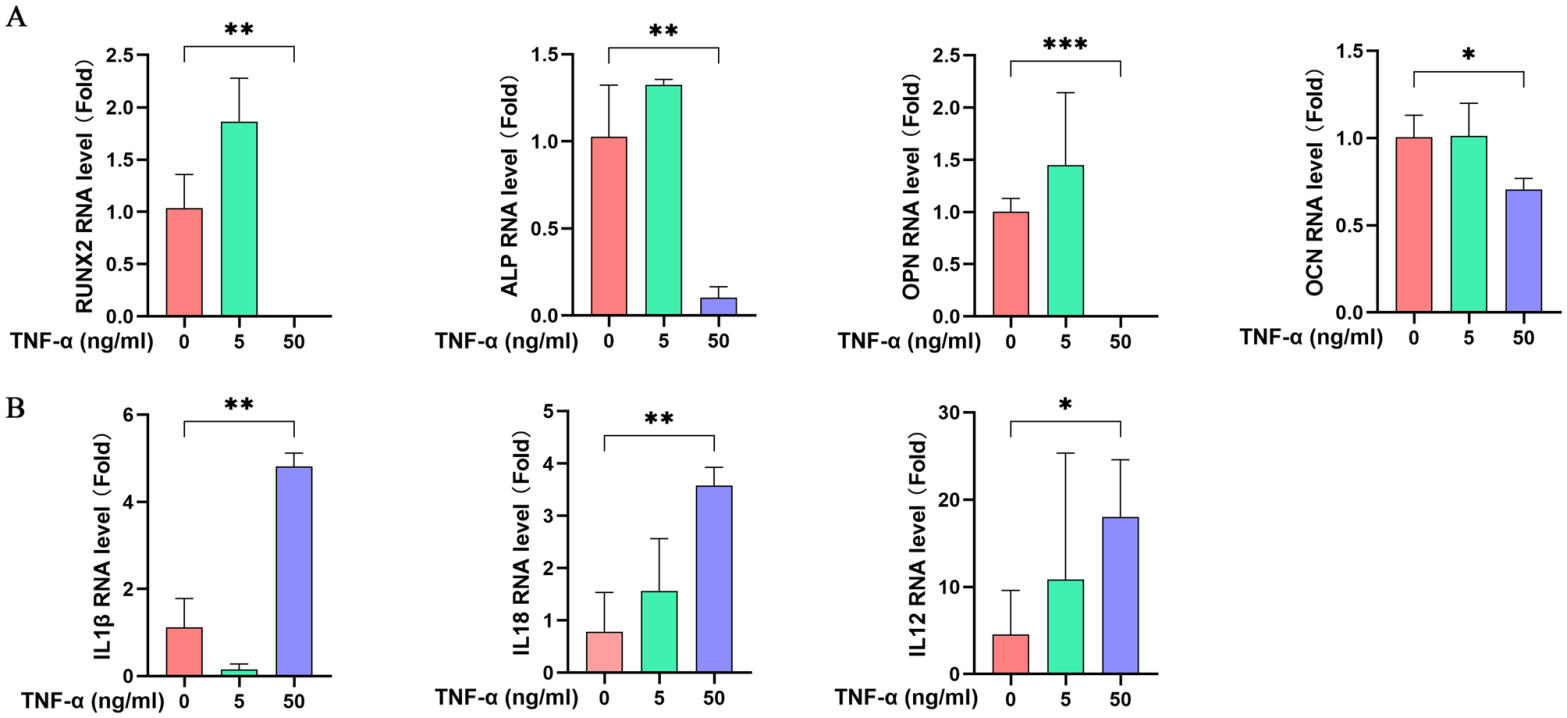
Inhibition of osteoblast differentiation under TNF-α stimulation and increased expression of inflammation-related genes. Osteogenic differentiation-related genes (**A**) and pro-inflammation-related genes (**B**)

After treatment with TNF-α, RT-PCR was used to detect RNA expression of key genes related to osteogenic differentiation and inflammation. As shown in Fig. 8, TNF-α treatment significantly decreased the expression of osteogenic differentiation-related genes in MC3T3-E1 cells, while the expression of inflammation-related genes increased with TNF-α concentration. These results indicate that TNF-α simulation impairs osteogenic differentiation and enhances the inflammatory environment in MC3T3-E1 cells.

## Discussion

Our comprehensive analysis, combining bioinformatics, bibliometric data, and experimental results, revealed a significant association between MIR17HG and osteoblast differentiation and death.​ Functional enrichment analysis indicated that MIR17HG-related mRNAs are involved in apoptosis and necroptosis, with key signaling pathways regulating cell death and inflammation. Under TNF-α stimulation, both MIR17HG and PANoptosis-related genes showed increased expression, accompanied by STAT3 phosphorylation. These findings suggest that MIR17HG may promote apoptosis in osteoblasts during osteogenic differentiation when stimulated by TNF-a.

PANoptosis, a newly identified type of PCD that includes apoptosis, pyroptosis, and necroptosis, appears to be enhanced in osteoblasts under inflammatory conditions. However, the interaction between PANoptosis and osteoblast differentiation in inflammatory settings remain underexplored. There is substantial evidence that abnormal lncRNA expression affects osteoblast proliferation, differentiation, and death [30–32], but its role in PANoptosis is unclear. Our objective was to establish a connection between PANoptosis, osteogenic differentiation, inflammation, and lncRNAs in order to understand the regulatory roles of PANoptosis-related and inflammation-related lncRNAs in the process of osteogenic differentiation.

By integrating cell sequencing data with publicly available transcriptome datasets, we identified MIR17HG as an lncRNA that correlates with PANoptosis-related proteins in osteoblasts under inflammatory conditions. Our ceRNA network and subsequent GO/KEGG analyses suggested that MIR17HG is associated with osteoblast differentiation and death, implicating pathways such as PI3K-Akt, MAPK, JAK-STAT, and TNF. Notably, MIR17HG and the miR-17-92 cluster promoter contain potential STAT3 binding sites [33–35]. The miR-17-92 cluster regulates the JAK2-STAT3 signaling pathway, with miR-18a and miR-19a promoting STAT3 activity and phosphorylation, respectively [36–38]. The JAK2/STAT3 pathway mediates proliferation and apoptosis in various cells [39, 40] and negatively regulates osteoblast differentiation [41, 42]. This evidence indicates a correlation between MIR17HG and PANoptosome occurrence in osteoblasts, suggesting that MIR17HG may regulate STAT3 phosphorylation and thus PANoptosis during osteogenic differentiation.

PANoptosis is a newly defined form of cell death relevant to immune response-related diseases. It is regulated by the multimeric protein complex PANoptosome [43], which influences pyroptosis, apoptosis, and necroptosis. Each of these processes has been studied separately by many researchers. Studies on PANoptosis suggest that CASP1, which drives pyroptosis, CASP8, which drives apoptosis, and RIPK1 and RIPK3, which drive necroptosis, can form a PANoptosome with other components [19]. This is consistent with our bibliometric data, where keyword visualization analyses showed high frequencies of CASP1, CASP8, RIPK1, and RIPK3 (Table 3). Our bibliometric analysis also shows that the NLRP3 inflammasome is mentioned in the context of pyroptosis, apoptosis, and necroptosis, acting as a sensor to regulate these cell death modalities. Pyroptosis is mediated by gasdermin family members via inflammasome-mediated caspase-1 cleavage of gasdermin D (GSDMD), caspase-11/4/5- or caspase-8-mediated cleavage of GSDMD, or caspase-3-mediated cleavage of gasdermin E (GSDME) [44–47]. Apoptosis is induced by caspase-3 and -7 following\activation of upstream initiator caspases caspase-8/10 or -9 [48, 49]. Necroptosis is induced by RIPK3-mediated MLKL oligomerization [50–52]. Our experimental data show that the expression of NLRP3 and genes associated with these cell death types increased in response to TNF-α stimulation. Therefore, we hypothesized that the NLRP3 inflammasome serves as an upstream signal regulating the onset of osteoblast apoptosis.

**Table 3:** The occurrences and link strength of PANoptosis-related proteins in keyword visualization analysis

Label	Total link strength	Occurrences
asc	42	3
caspase-1	145	13
caspase-3	56	4
caspase-7	56	4
caspase-8	188	19
caspases	79	11
gsdmd	288	34
nlrp3 inflammasome	273	32
ripk1	75	7
ripk3	195	19
zbp1	57	4
mlkl	148	16
fadd	24	4

**Fig. 9:**
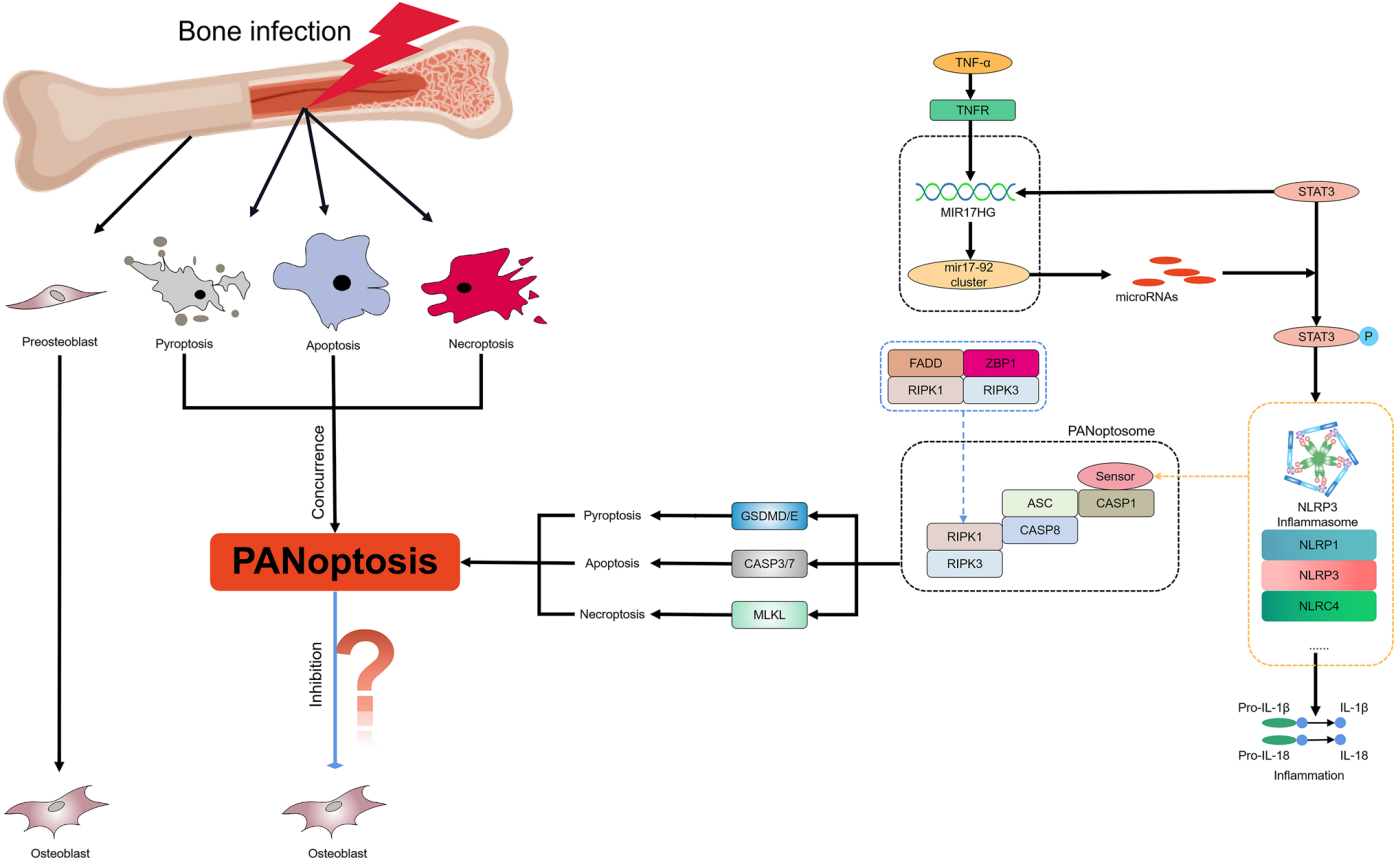
Under TNF-α stimulation, which activated STAT3 activity and phosphorylation. STAT3 activation promoted MIR17HG expression and transcription into miR-17-92 clusters, while microRNAs produced by processing of miR-17-92 clusters enhanced STAT3 transcriptional activity and promoted STAT3 phosphorylation through different pathways. phosphorylation. Phosphorylated STAT3 regulation promotes NLRP3 expression and the formation of NLRP3 inflammasome vesicles, thus participating in PANoptosis

Bioinformatics evidence alone is insufficient to prove this hypothesis, therefore, we detected the expression of genes related to PANoptosis and the osteogenic differentiation-related gene ELP2 using RT-PCR and WB experiments. The results showed that MIR17HG expression was positively correlated with STAT3, ELP2, and PANoptosis-related genes, indicating that MIR17HG may directly or indirectly affect osteoblast differentiation and PANoptosis. We hypothesized that MIR17HG regulates PANoptosis during osteogenic differentiation. Upon TNF-α stimulation, MIR17HG expression increases, promoting miRNA transcription that activates and phosphorylates STAT3. MIR17HG may influence the onset of PANoptosis in osteoblasts during osteogenic differentiation by regulating STAT3 expression (Fig. 9). Furthermore, our study provides new insights into the mechanism of osteoblast apoptosis and innovative ideas for treating inflammation in bone infections and osteomyelitis. In summary, our study offers directions for further exploration of the underlying mechanisms of inflammation in bone infections.

However, our study has limitations. First, while we performed the construction and high-throughput sequencing of the inflammatory model, the sequencing data for normal osteogenic differentiated cells were obtained from the web-based GEO database. Second, we studied only one cell line, MC3T3-E1, which requires further validation in other cell lines. Third, our study only investigated the correlation between MIR17HG and the inhibition of osteogenic differentiation and PANoptosis in osteoblasts without investigating the specific mechanism of action; these mechanisms can be further developed and refined in future studies.

## Conclusion

In conclusion, our comprehensive bioinformatics, bibliometric, and several experimental validation studies have shown that that lncRNA MIR17HG is interrelated with PANoptosis and acts a pivotal part in osteoblast apoptosis under TNF- α stimulation by modulating inflammatory response pathways. These finding positions MIR17HG as a promising candidate for identifying inflammatory biomarkers and developing therapeutic targets in osteoarthritis, bone infections, and other bone-related inflammatory diseases. These results highlight the potential of MIR17HG in advancing our comprehension of the molecular processes involved in osteoblast differentiation and apoptosis in inflammatory environments. Future studies should focus on validating these findings in other cell lines and in vivo models to further elucidate the specific mechanism of MIR17HG’s regulatory role. Additionally, exploring the therapeutic potential of targeting MIR17HG in clinical settings could pave the way for novel treatments for inflammatory bone diseases.

## Supplementary Information

Below is the link to the electronic supplementary material.

Supplementary file1 (TIF 6889 KB)

Supplementary file2 (DOCX 15 KB)

Supplementary file3 (XLSX 506 KB)

Supplementary file4 (XLSX 30 KB)

## Data Availability

In the Article/Supplementary Material are the original contributions that were made and presented in the study. The appropriate author can be contacted for more information.

## Abbreviations

TNF-α: Tumor necrosis factor-alpha
lncRNA: Long non-coding RNA
MIR17HG: MiR-17-92a-1 cluster host gene
STAT3: Signal transducer and activator of transcription 3
ELP2: Elongator acetyltransferase complex subunit 2
NLRP3: NLR family pyrin domain containing 3
MC3T3-E1: Mouse embryo osteoblast precursor cells
PCD: Programmed cell death
miRNA: MicroRNA
SIRPA: Signal regulatory protein alpha
LPS: Lipopolysaccharide
IL: Interleukin
NO: Nitric oxide
MyD88: Myeloid differentiation factor 88
TRIF: TIR domain-containing adaptor molecule 2
MEM: Minimum essential medium
FBS: Fetal bovine serum
GEO: Gene expression omnibus
GO: Gene ontology
KEGG: Kyoto encyclopedia of genes and genomes
PCR: Polymerase chain reaction
WB: Western blotting
CASP: Cysteinyl aspartate specific proteinase
GSDMD: Gasdermin D
GSDME: Gasdermin E
RIPK: Receptor-interacting protein kinase
MLKL: Mixed lineage kinase domain like
FADD: Fas-associating protein with a novel death domain
JAK2: Janus kinase 2
ZBP1: Z-DNA binding protein 1
TUG1: Taurine up-regulated 1
PD: Parkinson’s disease
