# Supplementary figures and images for: Analysis of PANoptosis-related ceRNA network reveals lncRNA MIR17HG involved in osteogenic differentiation inhibition impaired by tumor necrosis factor-α
Source: Mol Biol Rep. 2024 Aug 15;51(1):909. doi: 10.1007/s11033-024-09810-0 (PMC11327206; doi:10.1007/s11033-024-09810-0)

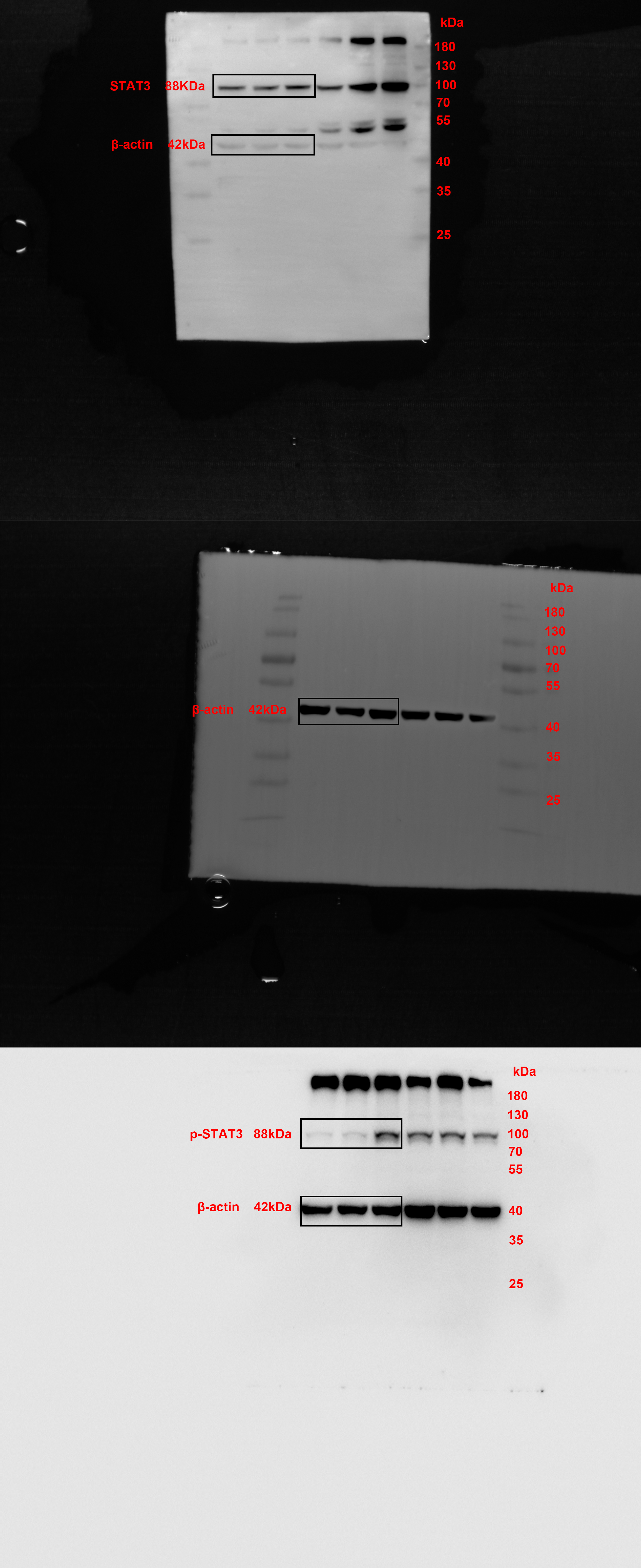

Supplement: Supplementary file 1 — Supplementary file1 (TIF 6889 KB) [file 11033_2024_9810_MOESM1_ESM.tif]
