# Supplementary material for: Analysis of PANoptosis-related ceRNA network reveals lncRNA MIR17HG involved in osteogenic differentiation inhibition impaired by tumor necrosis factor-α
Source: Mol Biol Rep. 2024 Aug 15;51(1):909. doi: 10.1007/s11033-024-09810-0 (PMC11327206; doi:10.1007/s11033-024-09810-0)
Supplement: Supplementary file 2 — Supplementary file2 (DOCX 15 KB) [file 11033_2024_9810_MOESM2_ESM.docx]

| miR-17-92 Forward Primer | 5`-CAGTAAAGGTAAGGAGAGCTCAATCTG-3` |
| --- | --- |
| miR-17-92 Reverse Primer | 5`-CATACAACCACTAAGCTAAAGAATAATCTGA-3` |
| MIR17HG Forward Primer | 5`-GCAGCACAGTTGGTTTCAGG-3` |
| MIR17HG Reverse Primer | 5`-TGGCAGGCTTCCTATGTTGG-3` |
| ELP2 Forward Primer | 5`-GCTGGAGGTGTCTCATGTGTT-3` |
| ELP2 Reverse Primer | 5`-GCACAACGGAGCAGGATGT-3` |
| NLRP3 Forward Primer | 5`-ATTACCCGCCCGAGAAAGG-3` |
| NLRP3 Reverse Primer | 5`-CATGAGTGTGGCTAGATCCAAG-3` |
| CASPASE 1 Forward Primer | 5`-ACAAGGCACGGGACCTATG-3` |
| CASPASE 1 Reverse Primer | 5`-TCCCAGTCAGTCCTGGAAATG-3` |
| CASPASE 3 Forward Primer | 5`-CTCGCTCTGGTACGGATGTG-3` |
| CASPASE 3 Reverse Primer | 5`-TCCCATAAATGACCCCTTCATCA-3` |
| CASPASE 8 Forward Primer | 5`-TGCTTGGACTACATCCCACAC-3` |
| CASPASE 8 Reverse Primer | 5`-GTTGCAGTCTAGGAAGTTGACC-3` |
| CASPASE 7 Forward Primer | 5`-AAGACGGAGTTGACGCCAAG-3` |
| CASPASE 7 Reverse Primer | 5`-CCGCAGAGGCATTTCTCTTC-3` |
| CASPASE 9 Forward Primer | 5`-GGCTGTTAAACCCCTAGACCA-3` |
| CASPASE 9 Reverse Primer | TGACGGGTCCAGCTTCACTA-3` |
| MLKL Forward Primer | 5`-TCGATTCTCCCAACATCTTGC-3` |
| MLKL Reverse Primer | 5`-GGTGTAGCCTGTATAAGCCTCTG-3` |
| GSDME Forward Primer | 5`-TGCAACTTCTAAGTCTGGTGACC-3` |
| GSDME Reverse Primer | 5`-AGTCTGACTCCACAACCACTG-3` |
| GSDMD Forward Primer | 5`-CCATCGGCCTTTGAGAAAGTG-3` |
| GSDMD Reverse Primer | 5`-ACACATGAATAACGGGGTTTCC-3` |
| RIPK1 Forward Primer | 5`-GACAGACCTAGACAGCGGAG-3` |
| RIPK1 Reverse Primer | 5`-CCAGTAGCTTCACCACTCGAC-3` |
| RIPK3 Forward Primer | 5`-CAGTGGGACTTCGTGTCCG-3` |
| RIPK3 Reverse Primer | 5`-CAAGCTGTGTAGGTAGCACATC-3` |
| GAPDH Forward Primer | 5`-AGGTCGGTGTGAACGGATTTG-3` |
| GAPDH Reverse Primer | 5`-GGGGTCGTTGATGGCAACA-3` |
| RUNX2 Forward Primer | GACTGTGGTTACCGTCATGGC-3` |
| RUNX2 Reverse Primer | 5`-ACTTGGTTTTTCATAACAGCGGA-3` |
| ALP Forward Primer | 5`-CCAACTCTTTTGTGCCAGAGA-3` |
| ALP Reverse Primer | 5`-GGCTACATTGGTGTTGAGCTTTT-3` |
| OPN Forward Primer | 5`-ATCTCACCATTCGGATGAGTCT-3` |
| OPN Reverse Primer | 5`-TGTAGGGACGATTGGAGTGAAA-3` |
| IL1B Forward Primer | 5`-GAAATGCCACCTTTTGACAGTG-3` |
| IL1B Reverse Primer | 5`-TGGATGCTCTCATCAGGACAG-3` |
| IL18 Forward Primer | 5`-GACTCTTGCGTCAACTTCAAGG-3` |
| IL18 Reverse Primer | 5`-CAGGCTGTCTTTTGTCAACGA-3` |
| IL12 Forward Primer | 5`-CAATCACGCTACCTCCTCTTTT-3` |
| IL12 Reverse Primer | 5`-CAGCAGTGCAGGAATAATGTTTC-3` |
| OCN Forward Primer | 5`-CTGACCTCACAGATCCCAAGC-3` |
| OCN Reverse Primer | 5`-TGGTCTGATAGCTCGTCACAAG-3` |
